# Immiscible Polymer Blends Made from Industrial Shredder Residue Mixed Plastic *with* and *without* Melt Blending

**DOI:** 10.1021/acsapm.4c00360

**Published:** 2024-05-24

**Authors:** Kanjanawadee Singkronart, Jussi Virkajärvi, Kristian Salminen, Siti Ros Shamsuddin, Koon-Yang Lee

**Affiliations:** ^†^Department of Aeronautics and ^‡^Institute for Molecular Science and Engineering (IMSE), Imperial College London, SW7 2AZ London, United Kingdom; § VTT Technical Research Centre of Finland Ltd, FI-40101 Jyväskylä, Finland

**Keywords:** polymer blending, fracture toughness, microtomography, extrusion, injection molding

## Abstract

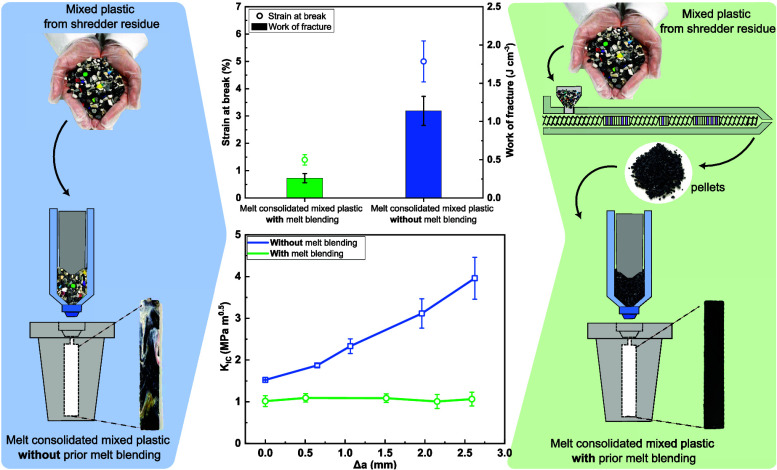

The processing of an immiscible polymer blend using melt blending (i.e., extrusion) often results in a polymer material with inferior mechanical performance compared with its virgin counterparts. Here, we report and compare the properties of immiscible polymer blends produced from industrial mixed plastic waste from shredder residue comprising at least four different polymers (acrylonitrile butadiene styrene, polystyrene, polypropylene, and polyethylene) *with* and *without* a prior melt-blending step employed. As anticipated, mixed plastic blend produced *with* a prior melt-blending step exhibited a more homogeneous microstructure, resulting in brittleness, poor work of fracture, and single-edge notched fracture toughness with a flat R-curve. Without the intimate polymers mixing arising from melt blending, the resulting mixed plastic blend was found to possess a more heterogeneous concentric ellipsoid microstructure with large single polymer domains. This mixed plastic blend demonstrated progressive failure under uniaxial tensile loading, along with a more ductile single-edge notched fracture toughness response accompanied by a growing R-curve. Digital image correlation and fractographic analysis revealed that melt blending created a large number of incompatible polymer boundaries that acted as stress concentration points, leading to brittleness and earlier onset catastrophic failure. The more heterogeneous mixed plastic blend produced *without* using a prior melt-blending step contains a smaller number of incompatible polymer boundaries. Additionally, the presence of larger single polymer domains also implies that the mechanical characteristics of the single polymer can be exploited in the immiscible mixed plastic blend. Our work opens up a simple pathway to add value to mixed plastic waste from shredder residue for use in engineering applications, diverting them away from landfill or incineration.

## 1 Introduction

The European Union aims to achieve climate neutrality by 2050 as part of the European Green Deal.^1^ At the heart of this is the Circular Economy Action Plan.^2,3^ This Action Plan calls for the need to track and trace resources and wastes, reduce carbon footprints and single use products, and design more eco-friendly plastics for electronics, vehicles, and packaging. As a result of this governmental initiative and the public’s growing demand for more environmentally friendlier materials, we saw a significant increase in the recycling rate of plastics used in the packaging sector. In EU27, 46% of plastic packaging waste collected was successfully recycled in 2022,^4^ corresponding to ca. 8.2 Mt of plastic waste successfully diverted from landfill and incineration. Such high recycling rate, ranked just behind the recycling of paper, glass, and metals,^5^ is mainly driven by the fact that plastics used for packaging are predominantly made of single polymer (usually polypropylene, high-density polyethylene, or polyethylene terephthalate) and are well labeled.^6−9^ Therefore, consumers can easily segregate plastic packaging waste generated at home prior to collection for subsequent sorting and recycling in materials and polymer recovery facilities (MRFs/PRFs). However, the recycling rates of plastic waste arising from electrical and electronic equipment, as well as the automotive sector, are significantly lower.^10^ It has been reported that only 25% of the plastics collected (ca. 449 kt) from waste electrical and electronic equipment (WEEE) were successfully recycled. The plastic recycling rate of end-of-life vehicle (ELV) is even worse; only 19% of the plastics collected (ca. 274 kt) was successfully recycled. The main end-of-life options for these waste plastics (∼2.5 Mt per year combined) are landfill (ca. 42%) and incineration for energy recovery (ca. 58%), which emit ∼4.2 Mt of CO_2_ equivalent to the environment annually.^11^

A major barrier to increasing the recycling rates of plastic waste arising from WEEE and ELV is the relatively low value of plastics in these sectors.^4,12,13^ The recycling technologies employed to recover materials from WEEE and ELV are optimized to recover the metallic fraction, which is the more valuable fraction of the waste. Generally speaking, a WEEE or ELV is first dismantled to recover any immediately identifiable components for reuse (ca. 21% of the total mass).^14−17^ The remainder is then shredded and sorted, either magnetically or using an eddy current, to recover any remaining metallic fraction. The shredder residue, i.e., the nonmetallic fraction of the shredded waste, which may consist any combinations of polyurethane (PU) foam, textiles, glass, acrylonitrile butadiene styrene (ABS), polystyrene (PS), polycarbonate (PC), poly(methyl methacrylate) (PMMA), polyvinyl chloride (PVC), polypropylene (PP), and polyethylene (PE), is then disposed of through landfill or incineration.^12,15,16^ This is because additional sorting of this highly heterogeneous mixed plastic waste is challenging due to the similarity in density and conductivity of the individual polymer.^17^ Moreover, a variety of additives, such as pigments and flame retardants, are often used in these polymers.^12,13^ Proper labeling is also absent, and this complicates the identification of the individual polymers.

To divert this shredder residue mixed plastic waste from landfill and incineration, the easiest method is to repurpose it as it is.^18,19^ However, most polymers are incompatible and immiscible at the molecular level.^20−25^ Consequently, enthalpic repulsion between the different immiscible polymers becomes dominant, leading to poor adhesion at this boundary between the immiscible polymers and causing earlier onset failure. This produces a polymer product with inferior mechanical properties compared with their virgin counterparts. The Flory–Huggins Gibbs free energy of mixing (Δ*G*_mix_) of an immiscible polymer blend is written as^26,27^
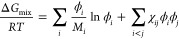
1where *R* is the universal gas constant, *T* is temperature, *M* is the degree of polymerization, and ϕ is volume fraction of the different polymers in the blend. The term χ_*ij*_ is the interaction parameter and can be estimated using
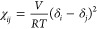
2whereby *V* is volume of mixing, and δ is the solubility parameter of the different polymers. A miscible polymer blend will form spontaneously if Δ*G*_mix_ ≤ 0. Since *M_i_* is orders of magnitude larger than ln ϕ_*i*_, Δ*G*_mix_ is predominantly governed by the magnitude of χ_*ij*_, which will always be ≥0. Only polymers with very similar δ values will yield a χ_*ij*_ ≈ 0 and a Δ*G*_mix_ ≤ 0. For a heterogeneous mixed plastic waste from shredder residue that contains very different polymers, the value of δ is sufficiently different (e.g., PP = 15 [J/mL]^1/2^ vs PMMA = 23 [J/mL]^1/2^)^21,28^ that Δ*G*_mix_ is expected to be greater than 0.

Instead of the more conventional melt-blending of different immiscible polymers to add value,^12,13^ we report in this work an alternative approach to upcycle heterogeneous industrial mixed plastic waste from shredder residue by directly melt-consolidating the different polymer granules in the mixed plastic *without* subjecting them to a prior melt-blending step. Without the intimate polymer mixing arising from melt blending (in an extruder, for example), the contact between the different immiscible and incompatible polymers can be minimized, thereby reducing the deterioration in mechanical performance of the resulting immiscible polymer blend. This will increase the value of the mixed plastic waste from shredder residue and create a stronger demand for it to be used in engineering applications, diverting it away from landfill or incineration. This present work compares and discusses the morphology of melt-consolidated industrial mixed plastic waste from shredder residue *with* and *without* subjecting them to a prior melt bending step. The tensile and fracture toughness responses of these melt-consolidated mixed plastics are reported.

## 2 Materials and Methods

### 2.1 Materials

Industrial mixed plastic granules from shredder residue were kindly supplied by Axion Polymers (Manchester, U.K.). Based on the data provided, this batch of mixed plastic composed of 40–50 wt % acrylonitrile butadiene styrene (ABS), 30–40 wt % polystyrene (PS), 10–15 wt % polypropylene (PP), 3 wt % rubber, 2 wt % polyethylene (PE), and the remainders are unidentifiable. The different types of granules had been previously hand sorted and characterized based on their color and rigidity.^19^ The mean diameter (*D*_50_) of the granules was found to be 4 mm by using a sieving tower.

### 2.2 Processing of Industrial Mixed Plastic *with* and *without* Prior Melt Blending

**Figure 1:**
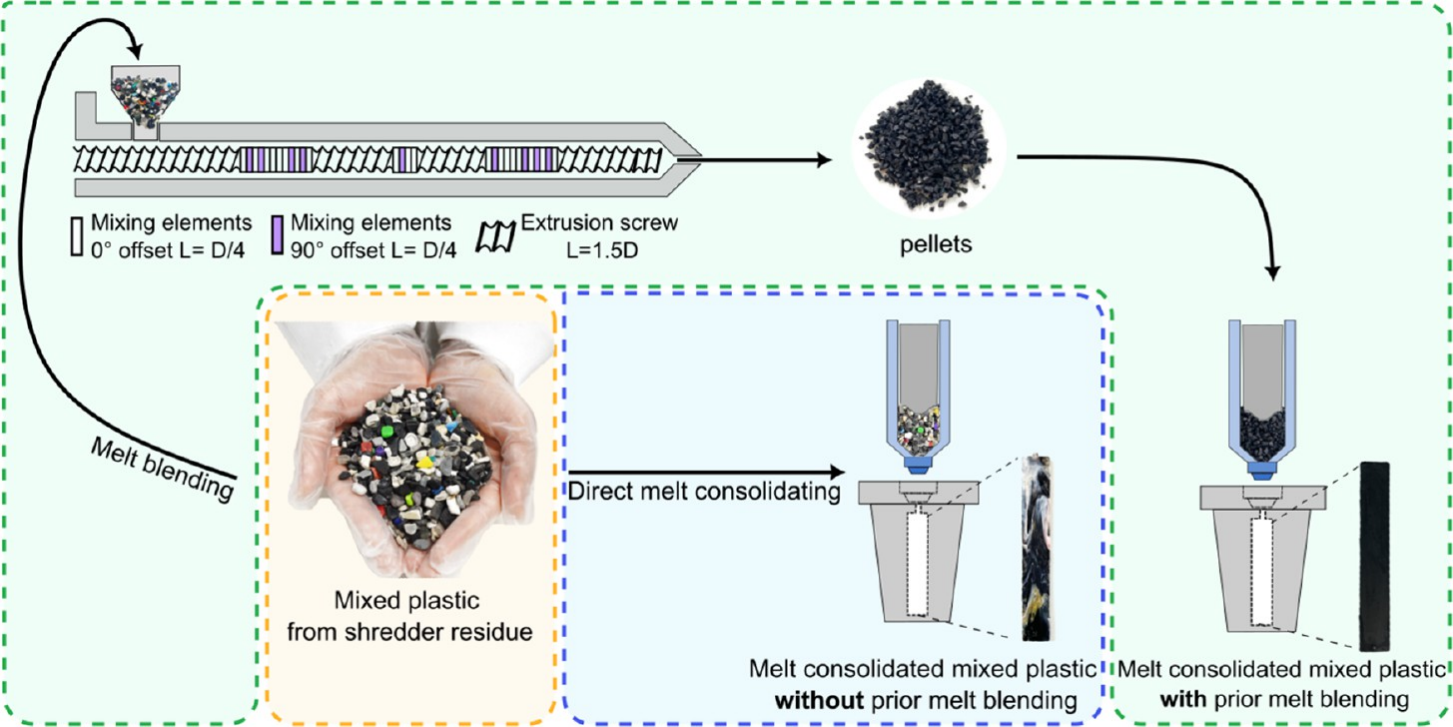
Schematic diagram summarizing the manufacturing of melt-consolidated mixed plastic from shredder residue *with* and *without* a prior melt-blending step.

Figure 1 summarizes the fabrication of melt-consolidated industrial mixed plastics from shredder residue *with* and *without* using a prior melt-blending step. To produce melt-consolidated mixed plastic blend *with* prior melt blending, the mixed plastic granules were first fed into a corotating twin-screw extruder (Eurolab XL, screw diameter = 16 mm, *L*/*D* = 25, Thermo Fischer Scientific, Karlsruhe, Germany). The profile of the screws is also illustrated in Figure 1. A processing temperature of 210 °C and a screw speed of 30 rpm were used based on our previous study.^19^ After melt blending, the extrudate was then pelletized (Haake VariCut, Thermo Fischer Scientific, Karlsruhe, Germany) and fed into a piston injection molding system (Haake MiniJet Pro, Thermo Fischer Scientific, Karlsruhe, Germany). To produce melt-consolidated mixed plastic blend *without* using a prior melt-blending step, the granules were fed directly into the piston injection molding system. All samples were injection molded into dog bone-shaped and rectangular test specimens. The barrel and mold temperatures of the injection molder were set to be 210 and 40 °C, respectively. The test specimens were injected at 650 bar for 10 s and held at the same pressure for another 60 s. The dog bone test specimen possessed an overall length of 65 mm, a thickness of 3 mm, a gauge length of 10 mm, and the narrowest part of the dog bone specimen was also 3 mm. The rectangular test specimen possessed an overall length of 80 mm, a width of 13 mm, and a thickness of 3 mm.

### 2.3 Characterization of Melt-Consolidated Industrial Mixed Plastic Blends Produced *with* and *without* Using a Prior Melt-Blending Step

#### 2.3.1 Microstructure of Melt-Consolidated Mixed Plastic Blends

X-ray microcomputed tomography (μCT) was used to investigate the microstructure of the melt-consolidated mixed plastic blends. Tomographic imaging of rectangular test specimens was conducted using an RX Solutions DeskTom 130 microtomography scanner (RX Solutions, Chavanod, France). Of the two scans performed, the first included two specimens fabricated *without* a prior melt-blending step, and the second included two specimens fabricated *with* a prior melt-blending step. Before imaging, the specimens were taped together and placed in vertical position on the sample holder. In the first (second) scan, the X-ray tube voltage was 40 kV (45 kV), the current was 200 μA (177 μA), the single frame exposure time was 1.18 s (1 s), the image pixel/voxel size was 12 μm (9.5 μm), and the volume of each sample captured in the image was around 13 mm × 3 mm × 16 mm (13 mm × 3 mm × 13 mm). Two frames were averaged for each projection image. Both scans covered a 360° rotation, including 1440 projection images. Reconstruction was done with RX Solutions X-Act software utilizing the filtered back projection algorithm. ImageJ software (version 13.0.6) was used in the visualizations.

#### 2.3.2 Scanning Electron Microscopy (SEM) of Melt-Consolidated Mixed Plastic Blends

SEM (TESCAN MIRA scanning electron microscope, Cambridge, U.K.) was used to further investigate the internal morphology of the samples. An accelerating voltage of 10 kV was used. Prior to SEM, the samples were sputter coated with Cr (Q150T ES, Quorum, East Sussex, United Kingdom) using a coating current of 120 mA for 210 s.

#### 2.3.3 Tensile Properties of Melt-Consolidated Mixed Plastic Blends

Tensile test was conducted in accordance with ASTM D638–14 using a universal testing machine (Model 4502, Instron, High Wycombe, U.K.) equipped with a 10 kN load cell. A crosshead displacement of 1 mm min^–1^ (corresponding to a strain rate of 0.1% s^–1^) was employed. Prior to the test, a speckle pattern was painted onto the surface of the dog bone-shaped test specimens. Digital image correlation (ARAMIS 12M, GOM UK Ltd., Coventry, U.K.) was then used to obtain the full strain field of the specimens during uniaxial tensile loading. An average of five specimens were tested for each type of sample.

#### 2.3.4 Single-Edge Notched Fracture Toughness of Melt-Consolidated Mixed Plastic Blends

The fracture toughness of the samples was determined from single-edge notch beam (SENB) test specimens loaded in three-point bending mode in accordance with ASTM5045–14. The support span length and the crosshead displacement speed used were 50 mm and 1 mm min^–1^, respectively. A sharp notch with a depth of 6.2 mm was introduced at half length from the edge of the specimen toward the center using a band saw (Startrite 502S, A.L.T. Saws & Spares Ltd., Kent, U.K.). The notch was further sharpened with a surgical scalpel prior to the SENB test. The initial crack length (*a*) to width (*w*) ratio, *x*, of the SENB test specimen was ∼0.49. The initial critical stress intensity factor, *K*_IC_, of the specimen was calculated from
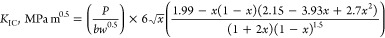
3where *P* is the load at crack initiation and *b* is the thickness of the test specimen. A speckle pattern was also painted onto the surface of the test specimens to track the strain field around the crack during fracture toughness testing by using digital image correlation.

## 3 Results and Discussion

### 3.1 Microstructure of Melt-Consolidated Industrial Mixed Plastic Blends Produced *with* and *without* Using a Prior Melt-Blending Step

**Figure 2:**
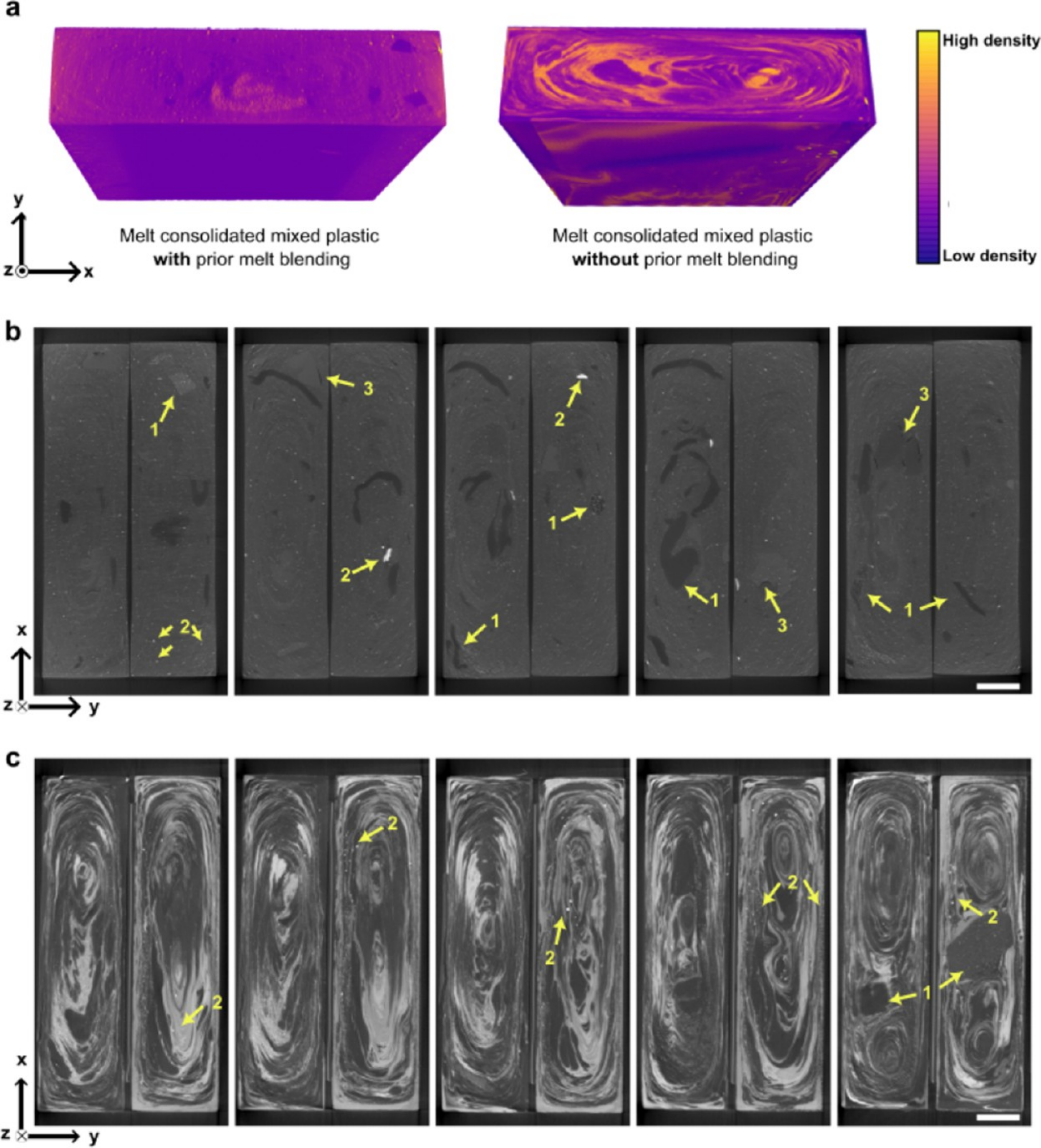
(a) 3D reconstruction of the melt-consolidated industrial mixed plastics *with* (left) and *without* (right) prior melt blending. The pseudocolor gradient represents local variations in material density, with yellow as higher density and purple as lower density. Horizontal cross-sectional slices of through the thickness of the melt-consolidated industrial mixed plastics (b) *with* prior melt blending and (c) *without* prior melt blending. Scale bar = 1.5 mm. See Section 3.1 for labels 1–3.

Figure 2a shows the 3D reconstructed melt-consolidated mixed plastic blends *with* and *without* prior melt-blending obtained using μCT. The pseudocolor contrast here denotes the density variation depicting the phase distribution of different materials. The horizontal cross-sectional slices along the length of the two types of mixed plastic blends are presented in Figure 2b (*with* prior melt blending) and Figure 2c (*without* prior melt blending). It can be seen from these figures that the mixed plastic blend produced *with* a prior melt-blending step employed possessed a smaller variation in material density distribution compared to the mixed plastic blend produced *without* using a prior melt-blending step, indicating that melt blending leads to a more homogeneous immiscible polymer blend. This stems from the high shear mixing during melt blending,^29,30^ whereby the different immiscible polymers were broken up and blended in the molten state throughout the material. Nevertheless, some inclusions can still be observed (Figure 2b). These can be attributed to the presence of nonmeltable polymers (see label 1), such as vulcanized rubber in the starting shredder residue mixed plastic waste, as well as inorganic fillers (white speckles, see label 2). The presence of inorganic fillers is not surprising as CaCO_3_, ZnO, Cr_2_O_3_, Fe_2_O_3_, and TiO_2_ are commonly used as additives for polymers in electrical and electronic equipment, as well as in vehicles.^31−33^ Cracks can also be observed around some of the nonmeltable polymers (see label 3), which could lead to earlier onset failure of this material (see Sections 3.3 and 3.4 later).^34^

Mixed plastic blends produced *without* using a prior melt-blending step possessed a large variation in material density distribution, which appeared in the form of concentric ellipsoids (i.e., deformed “onion-like” structures). The formation of such heterogeneous microstructure is hypothesized to be a combination of (i) the absence of intimate polymer mixing, (ii) the difference in melt rheology of the polymers during processing (PP and PE melt at ∼165 and ∼110 °C, respectively,^19,35^ while ABS and PS are amorphous), and (iii) the characteristic fountain flow arising from the injection molding process.^29,34^ Due to the absence of intimate polymer mixing, large molten single polymer domains with a size corresponding to one or more granules (see Figure 1 for example) are expected to form in the barrel of the injection molder. During the injection molding process, the molten material experienced shear and elongational stresses.^29,34,36,37^ At the nozzle exit and inside the mold, the pressure exerted caused the molten material to move in the radial direction, forming concentric ellipsoids. Nonmeltable polymers (label 1) and white speckles that corresponded to inorganic fillers (label 2) can also be observed. However, the inorganic fillers appeared to be contained within their respective single polymer domains instead of uniformly distributed throughout this material as seen in Figure 2b for the more homogeneous mixed plastic blend produced *with* prior melt blending.

### 3.2 Cryo-Fractured Surface of Melt-Consolidated Industrial Mixed Plastic Blends *with* and *without* Prior Melt-Blending

**Figure 3:**
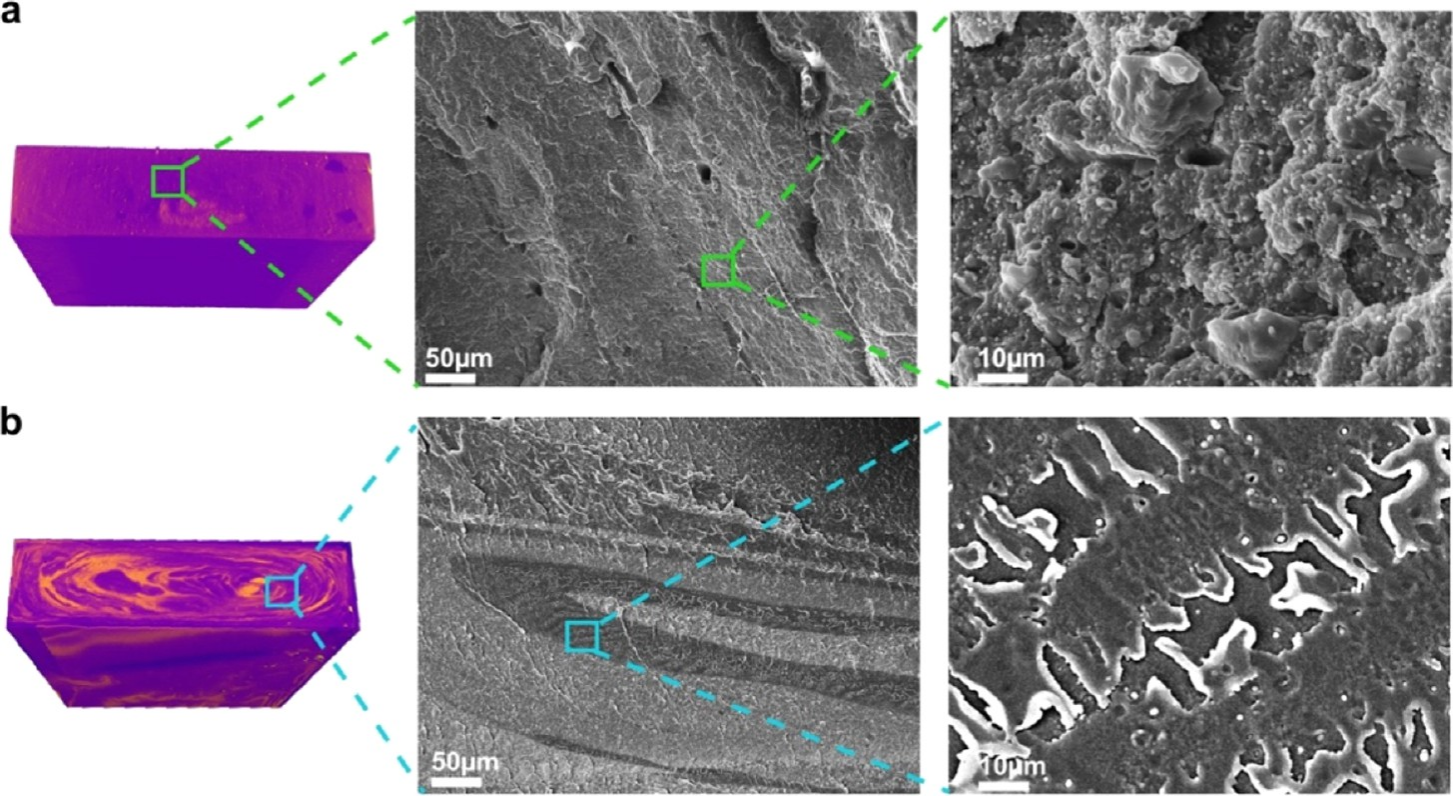
Cryo-fractured SEM images showing the internal morphology of melt-consolidated industrial mixed plastics produced (a) *with* a prior melt-blending step and (b) *without* using a prior melt-blending step.

The internal morphology of the mixed plastic blends is further investigated using SEM (see Figure 3). Conventionally when two immiscible polymers are melt-blended, a sea–island or cocontinuous morphology will be observed, depending on the weight fraction of the constituents.^18,20,38−41^ During melt blending, the minor phase is dispersed as spherical droplets in a continuous phase, forming the sea–island morphology. When the compositions of the constituents are similar, neither of them will form the dispersed phase. Instead, they will collide and deform into irregular shapes, creating a cocontinuous morphology. However, the SEM images presented in Figure 3a revealed that the more homogeneous mixed plastic blend produced *with* a prior melt-blending step possessed an ill-defined morphology that is neither a sea–island nor cocontinuous. A concentric ellipse morphology can be observed in the more heterogeneous mixed plastic blend produced *without* using a prior melt-blending step (see Figure 3b). This morphology corroborates those obtained using μCT shown in section 3.1. Each lamella observed is postulated to correspond to a single polymer. Fingering can also be observed at the interface between two lamellae. This is attributed to the instability of the interface due to the immiscibility and incompatibility between the polymers.^42,43^ The presence of inorganic fillers in these blends can also be seen in the SEM images.

### 3.3 Tensile Properties of Melt-Consolidated Industrial Mixed Plastic Blends *with* and *without* Prior Melt Blending

**Figure 4:**
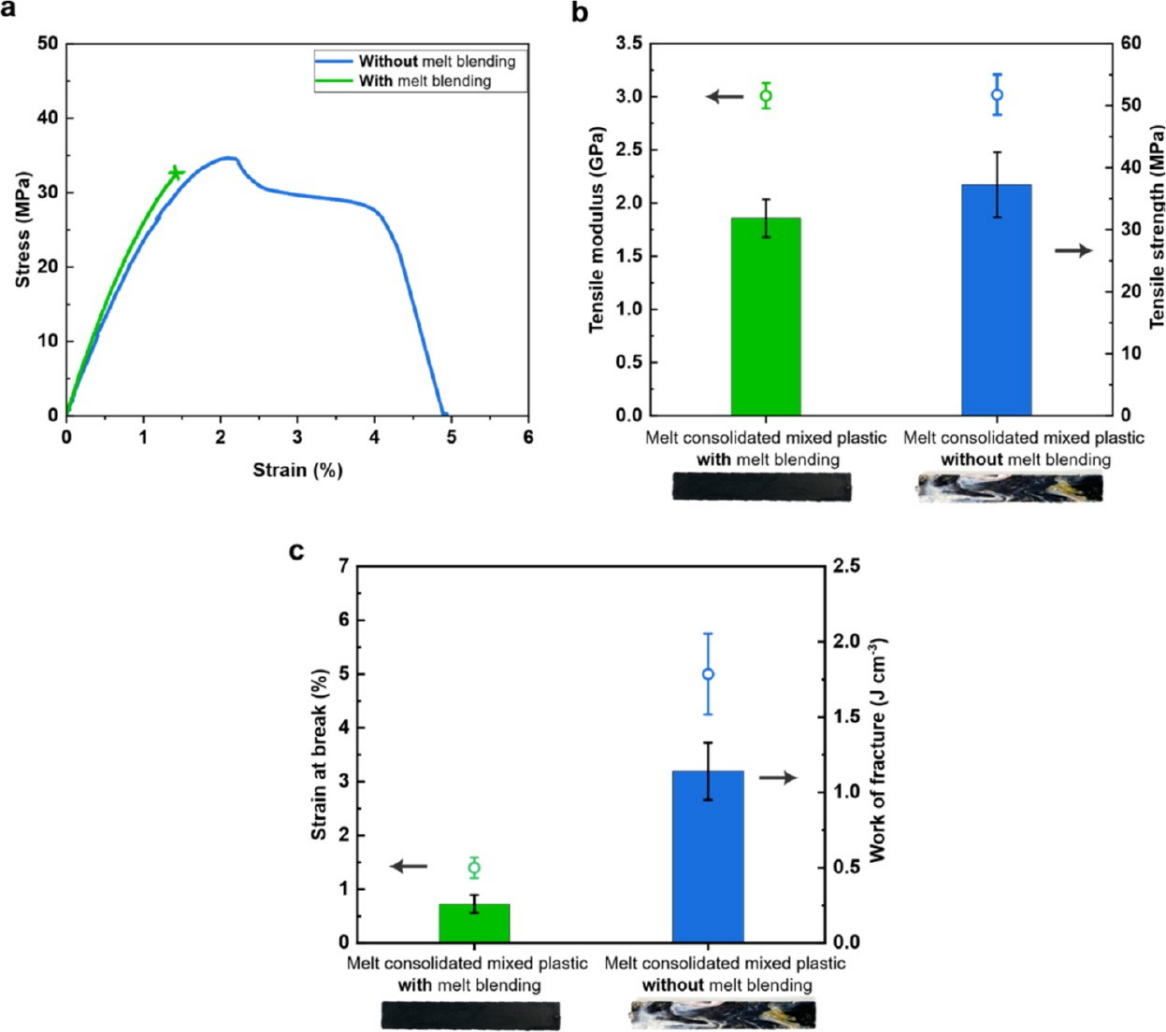
(a) Representative tensile stress–strain curves, (b) tensile modulus and strength as well as (c) tensile strain-at-break and work of fracture of the melt-consolidated industrial mixed plastics *with* and *without* prior melt blending.

Figure 4 presents the tensile properties of the mixed plastic blends produced *with* (green curve, column and icon) and *without* (blue curve, column and icon) using a prior melt-blending step. After the initial linear elastic response, mixed plastic blend produced with a prior melt-blending step fractured catastrophically, characterized by a sharp decrease in stress to zero when maximum load was reached (see Figure 4a). Such uniaxial tensile stress–strain response of immiscible polymer blend is not surprising. Mahanta et al.^44^ melt-blended ABS and PC, which are immiscible. The strain-at-break of neat ABS was 51% and neat PC was 8% but the melt blending of ABS and PC produced an inferior polymeric product with a strain-at-break of only 2%. Similar stress–strain response was also observed for other immiscible binary polymer blends, including PP/ABS,^45^ PS/PP,^21^ and PET/HDPE.^46^ Moreover, the mechanical performance deteriorates even more severely with increasing number of different polymers in the immiscible blend.^22,47^ Melt-blending only 5 wt % PP into an immiscible PC/PS blend led a 4-fold decrease in strain-at-break compared to the PC/PS blend without PP.^47^ Mixed plastic blend produced *without* a prior melt-blending step, on the other hand, underwent a progressive failure that is characterized by a gradual decrease in load-bearing capacity. Both materials were found to possess a similar tensile modulus of ∼3 GPa (see the hollow icons in Figure 4b). This is because the tensile modulus of a polymer blend is strongly dependent on the composition and volume fraction of the constituents.^48,49^ As both mixed plastic blends possess a similar polymer composition, the tensile moduli are, as expected to be, similar.

Nonetheless, the mixed plastic blend produced *without* using a prior melt-blending step was found to possess a higher tensile strength compared to those produced *with* a prior melt-blending step (see columns in Figure 4b). More importantly, the mixed plastic blend produced *without* using a prior melt-blending step possessed a higher strain-at-break (see hollow icons in Figure 4c) than that *with* a prior melt-blending step (5% vs 1.5%). It also follows that the tensile work of fracture, defined as the area under the tensile stress–strain curve, is higher for the mixed plastic blend manufactured *without* using a prior melt-blending step (1.14 J cm^–3^ compared to 0.26 J cm^–3^ for the mixed plastic blend produced *with* prior melt blending). It must be mentioned at this point that virgin PP, PE, ABS, and PS, which are the major constituents of this batch of mixed plastic, possess a tensile strain-at-failure and work of fracture of 578% and 38 J cm^–3^, 416% and 40 J cm^–3^, 77% and 7.5 J cm^–3^, as well as 5% and 2 J cm^–3^, respectively.^19^ The lower tensile work of fracture of the mixed plastic blends compared to their virgin counterparts is attributed to the incompatibility between the different polymers, with Δδ_PS/PP_ = 5.3 (J/mL)^0.5^, Δδ_ABS/PP_ = 3.8 (J/mL)^0.5^, Δδ_ABS/PS_ = 2.0 (J/mL)^0.5^, and Δδ_PS/PE_ = 4.4 (J/mL)^0.5^. As a result, Δ*G*_mix_ > 0 and the enthalpic repulsion between the different immiscible polymers causes deterioration in mechanical performance compared to their virgin counterparts.

### 3.4 Strain Field of Melt-Consolidated Industrial Mixed Plastic Blends *with* and *without* Prior Melt Blending During Uniaxial Tensile Loading

**Figure 5:**
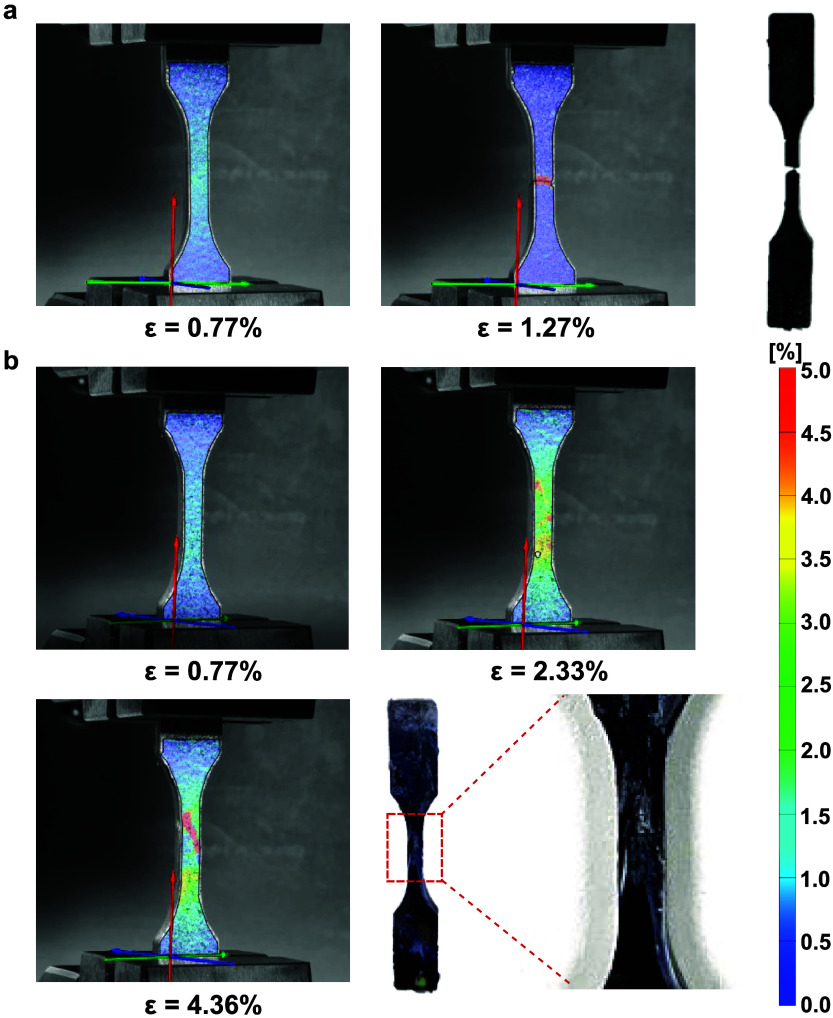
Full strain map of the samples during uniaxial tensile testing obtained from digital image correlation. (a) Melt-consolidated industrial mixed plastics produced *with* a prior melt-blending step and (b) melt-consolidated industrial mixed plastics produced *without* prior melt blending.

To ascertain why melt blending led to a lower tensile strength, strain-at-failure, and work of fracture, digital image correlation (DIC) was used (see Figure 5). The strain experienced by the mixed plastic blend produced *with* a prior melt-blending step employed was found to be uniformly distributed within the gauge length of the test specimen until a sudden fracture occurred at 1.27% strain. Such uniform strain distribution prior to fracture corroborates the homogeneity of the material (see Sections 3.1 and 3.2), whereby the melt-blending process blended the different immiscible polymers throughout the material. This also had the unfortunate consequence of creating a large number of incompatible polymer boundaries that acted as stress concentration points. Combining this with the presence of cracks around any nonmeltable polymers (see Section 3.1), earlier onset and catastrophic failure occurred. The more heterogeneous mixed plastic blend produced *without* using a prior melt-blending step contained larger single polymer domains due to the lack of intimate polymer mixing. It can therefore be inferred that the number of incompatible polymer boundaries is smaller. Furthermore, the presence of the larger single polymer domains also implies that the mechanical characteristics of the single polymer can be exploited. During uniaxial tensile deformation, these larger single polymer domains bore the stress before failure at an incompatible polymer boundary (Figure 5b). In fact, the origin of the failure can be traced back to the morphology of the specimen, where the crack was initiated from the boundary between two larger (incompatible) single polymer domains.

### 3.5 Uniaxial Tensile Fracture Surface of Melt-Consolidated Industrial Mixed Plastic Blends *with* and *without* Prior Melt Blending

**Figure 6:**
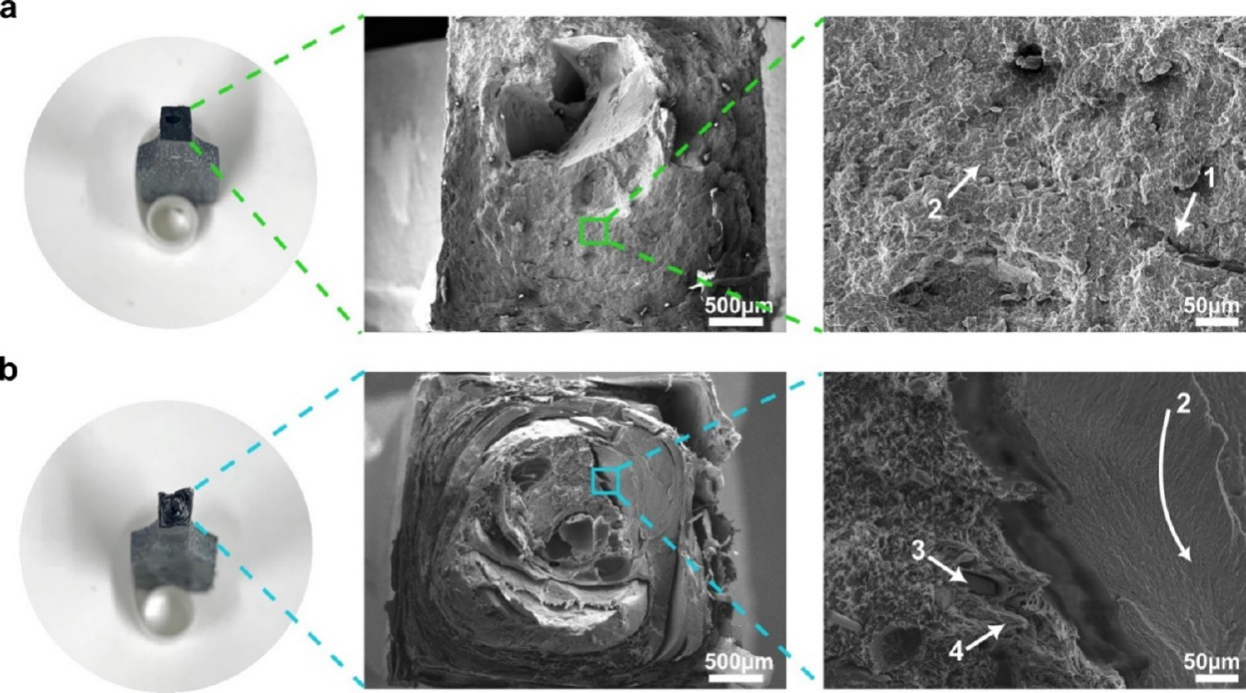
Tensile fracture surface of melt-consolidated industrial mixed plastics (a) *with* and (b) *without* prior melt blending. See Section 3.5 for label 1–4.

Fractographic analysis further revealed that the more homogeneous mixed plastic blend *with* prior melt blending exhibited scarps (Figure 6a, label 1), textured microflow (Figure 6a, label 2), and a distinct lack of plastic deformation features, all of which are characteristics of a brittle material.^50,51^ In contrast, the more heterogeneous mixed plastic blend fabricated *without* prior melt blending showed a single crack path along the boundary between two immiscible polymer domains (Figure 6b). The fracture surface also demonstrated a wide range of intrinsic fracture mechanisms corresponding to the respective single polymer in each domain. Textured microflow was found on one side of the fracture surface (Figure 6b, label 2) and the arrow denotes the direction of the propagating crack. This is a characteristic of a brittle polymer, which suggested that this particular polymer domain could be PS.^21,52^ The other side of the fracture surface showed polymer inclusion (Figure 6b, label 3) and fibrillation (Figure 6b, label 4). This thin fibril layer, accompanied by lateral contraction involving polymer chain mobility, is indicative of plastic deformation and has taken place in the direction of principal tensile stress. Such fracture feature is generally found in ductile polymers, suggesting that this polymer domain could be either ABS, PP, or PE.^40,53−56^ The presence of plastic deformation features translates to better mechanical performance of the more heterogeneous mixed plastic blend produced *without* melt blending.

### 3.6 SENB Fracture Toughness of Melt-Consolidated Industrial Mixed Plastic Blends *with* and *without* Prior Blending

**Figure 7:**
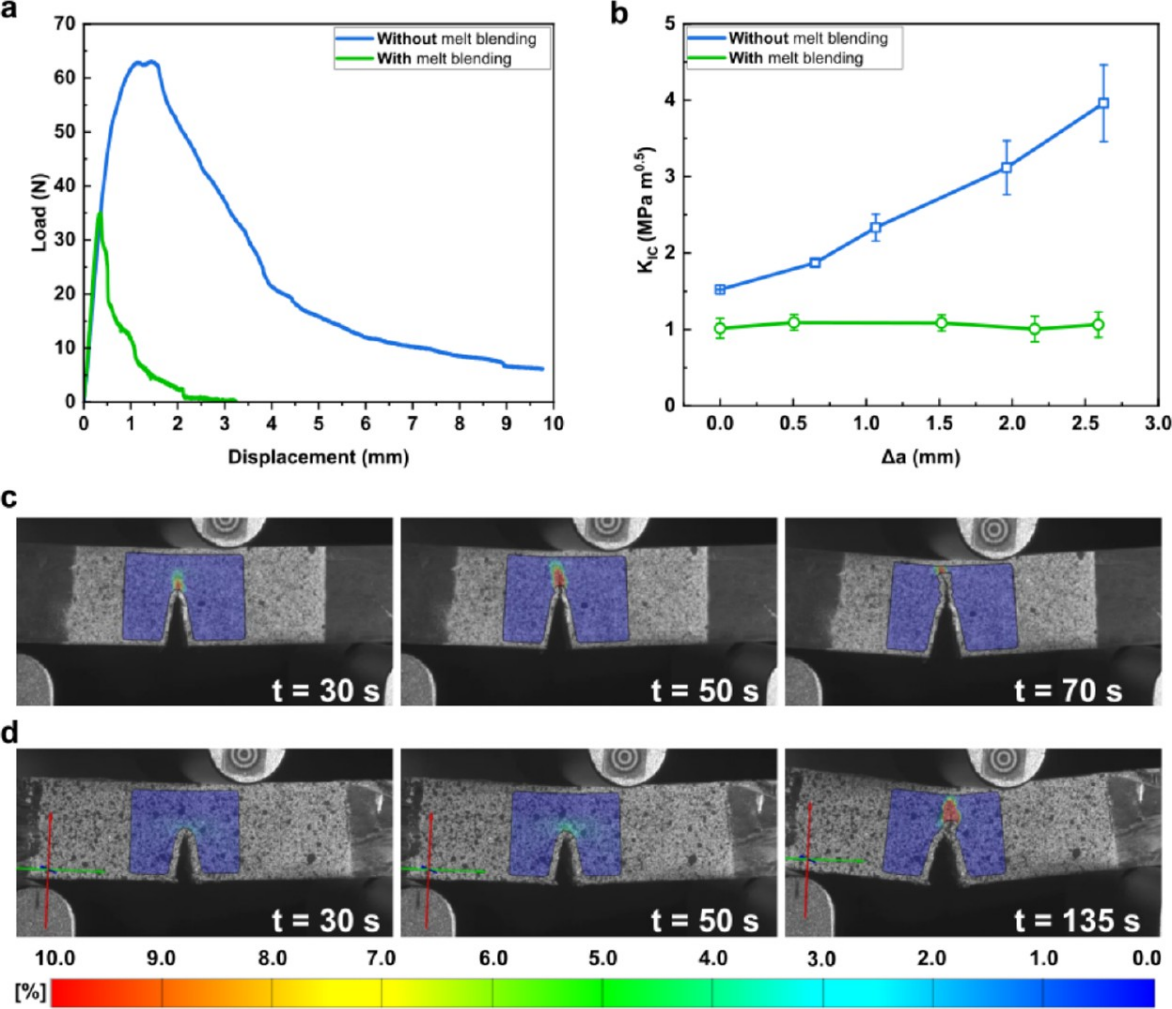
(a) Representative load–displacement and (b) R-curves of SENB melt-consolidated mixed plastic *with* and *without* prior melt blending. (c) Transverse strain of melt-consolidated mixed plastic *with* and (d) *without* prior melt blending.

Figure 7 summarizes the SENB fracture toughness response of the mixed plastic blends. Both materials exhibited a gradual load decrease after peak load was reached (Figure 7a). However, the more homogeneous mixed plastic blend produced *with* a prior melt-blending step was found to possess a lower SENB *K*_IC_ of only 0.94 MPa m^0.5^ and a flat R-curve (Figure 7b, green curve), indicative of poor crack resistance and brittleness of this material.^20,57^ The fracture energy was entirely dissipated inside a small plastic zone (Figure 7c). *Without* prior melt blending, the more heterogeneous mixed plastic blend possessed a higher SENB *K*_IC_ of 1.52 MPa m^0.5^, a 62% increase over the mixed plastic blend *with* prior melt blending. Moreover, it also possessed a growing R-curve (Figure 7b, blue curve). Essentially, it is more energetically costly to achieve each crack opening. It can be seen from Figure 7d that the plastic zone at the crack tip of this material increased in size as the crack propagated through, indicating an increase in energy dissipation through plastic deformation to sustain the crack growth (see section 3.7 later).^58^ This is thought to be due to the crack encountering the larger single polymer domain(s) that can undergo plastic deformation, or the crack could divert around it, leading to the improvement of fracture toughness. It must be mentioned however that the SENB *K*_IC_ of the mixed plastic blend *without* prior melt blending is still lower than its virgin counterparts. Virgin ABS, PP, PS, and HDPE possess SENB *K*_IC_ values of 2.74 MPa m^0.5^, 2.33 MPa m^0.5^, 2.24 MPa m^0.5^, and 1.68 MPa m^0.5^, respectively.

### 3.7 SENB Fracture Surface of Melt-Consolidated Industrial Mixed Plastic Blends *with* and *without* Prior Blending

**Figure 8:**
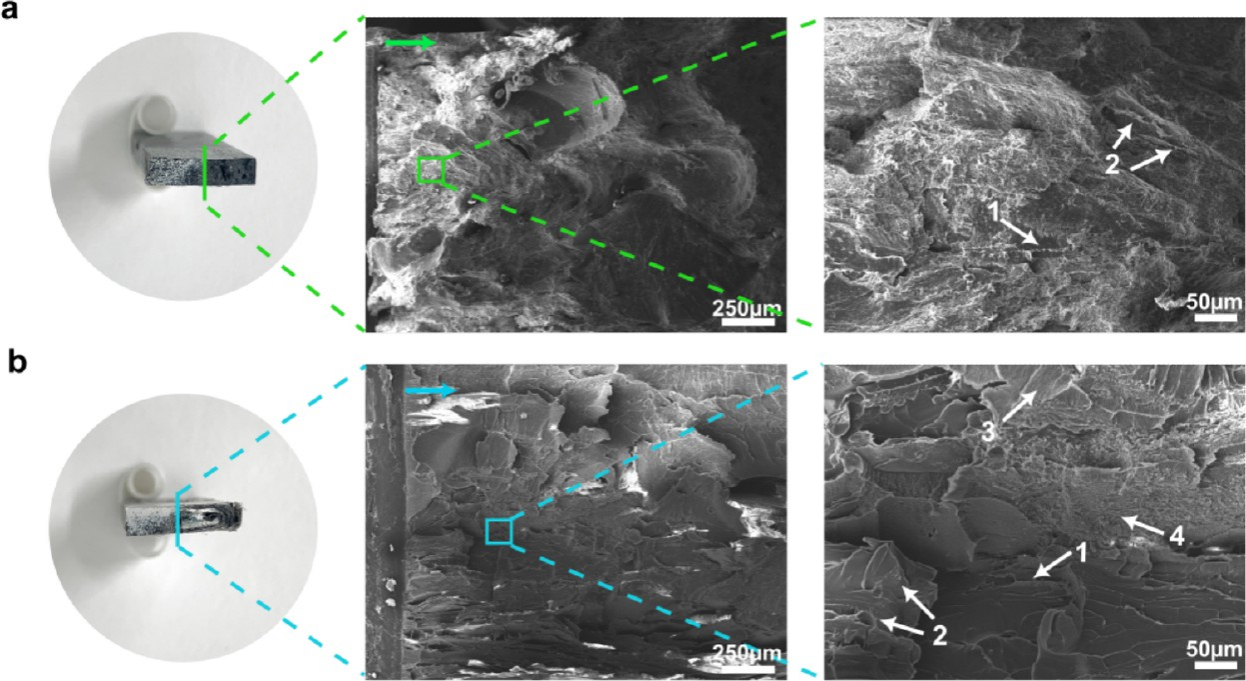
Fracture surface of SENB test specimens. (a) Melt-consolidated mixed plastic *with* and (b) *without* prior melt blending. The crack propagated from left to right. See Section 3.7 for label 1–4.

The SENB fracture surfaces of both mixed plastic blends are presented in Figure 8. It can be seen that the more homogeneous mixed plastic blend *with* prior melt blending exhibited localized cleavage and low level of yielding (Figure 8a). Under SENB loading, localized crack fronts overlap and coalesce, leading to features such as scarps (label 1) and riverline (label 2). Similar to the uniaxial tension fracture surface, the fracture surface of the more heterogeneous mixed plastic blend *without* prior melt blending also showed distinct fracture behavior of both brittle and ductile materials. The presence of riverline (Figure 8b, label 2), scraps (Figure 8b, label 1), and textured microflow (Figure 8b, label 3) indicated a brittle failure. This is postulated to stem from the PS domain. The observed fibrillation (Figure 8b, label 4) stems from a ductile polymer, such as ABS, PP, or PE, and it leads to extensive plastic deformation, giving rise to the observed growing R-curve (Figure 7b).

## 4 Conclusions

This study highlights the disadvantage of melt-blending immiscible polymers. *With* melt blending, the resulting mixed plastic blend possessed a more homogeneous microstructure but is also accompanied by a poor tensile strain-at-break (1.5%), work of fracture (0.26 J cm^–3^), SENB critical stress intensity factor (0.94 MPa m^0.5^), and a flat R-curve corresponding to brittle fracture. *Without* melt blending, the resulting mixed plastic blend possessed higher tensile strain-at-break of 5%, work of fracture of 1.14 J cm^–3^, SENB critical stress intensity factor of 1.52 MPa m^0.5^, and a growing R-curve with plastic deformation. The lack of intimate polymer mixing led to the formation of large single polymer domains that corresponded to the size of one or more mixed plastic granules. Consequently, the number of incompatible polymer boundaries is lower, and the mechanical characteristic of the single polymer can be better exploited by the resulting immiscible polymer blend. The principle explored here may also be applied in other types of mixed polymer waste such as plastic packaging waste that typically contains a mixture of PP, PE, PS, PVC, or multilayered structures, offering a strategy to diverting conventionally nonrecyclable polymers away from landfill and incineration.
